# Supplementary material for: Unified theory for light-induced halide segregation in mixed halide perovskites
Source: Nat Commun. 2021 May 11;12:2687. doi: 10.1038/s41467-021-23008-z (PMC8113520; doi:10.1038/s41467-021-23008-z)
Supplement: Supplementary file 1 — Supplementary Information [file 41467_2021_23008_MOESM1_ESM.pdf]

# Supplementary Information:
# Unified theory for light-induced halide segregation in mixed halide perovskites

Zehua Chen,[1,2] Geert Brocks,[1,2,3] Shuxia Tao,[1,2,*] and Peter A. Bobbert[2,4,†]

[1] *Materials Simulation and Modelling, Department of Applied Physics, Eindhoven University of Technology, 5600 MB Eindhoven, The Netherlands*

[2] *Center for Computational Energy Research, Department of Applied Physics, Eindhoven University of Technology, P.O. Box 513, 5600 MB Eindhoven, The Netherlands*

[3] *Computational Materials Science, Faculty of Science and Technology and MESA+ Institute for Nanotechnology, University of Twente, P.O. Box 217, 7500 AE Enschede, The Netherlands*

[4] *Molecular Materials and Nanosystems, Eindhoven University of Technology, P.O. Box 513, NL-5600 MB Eindhoven, The Netherlands*

(Dated: April 2, 2021)

* S.X.Tao@tue.nl
† P.A.Bobbert@tue.nl

## SUPPLEMENTARY NOTE 1. EQUILIBRIUM VOLUMES

In the quasichemical approximation (QCA) the equilibrium volumes per formula unit of the five compounds are obtained as $V(x,T) = \sum_j \bar{x}_j V_j$, where $\bar{x}_j$ is the thermal equilibrium fraction of microclusters with configuration $j$ (see "Methods" in the main text) and $V_j$ is the volume per formula unit of configuration $j$. The results at room temperature are shown in Supplementary Fig. 1. An increasing Br concentration leads to a decreasing volume due to the smaller ionic size of Br than I: $r_{\mathrm{I}} = 2.20$ and $r_{\mathrm{Br}} = 1.96$ Å[1, 2]. For the single-cation compounds we have $V_{\mathrm{FA}} > V_{\mathrm{MA}} > V_{\mathrm{Cs}}$, in accordance with the ionic sizes $r_{\mathrm{FA}} = 2.53$, $r_{\mathrm{FA}} = 2.17$, and $r_{\mathrm{Cs}} = 1.67$ Å[1, 2]. As expected, the partial Cs substitution in $\mathrm{MA}_{7/8}\mathrm{Cs}_{1/8}\mathrm{Pb}(\mathrm{I}_{1-x}\mathrm{Br}_x)_3$ and $\mathrm{FA}_{7/8}\mathrm{Cs}_{1/8}\mathrm{Pb}(\mathrm{I}_{1-x}\mathrm{Br}_x)_3$ decreases the volume.

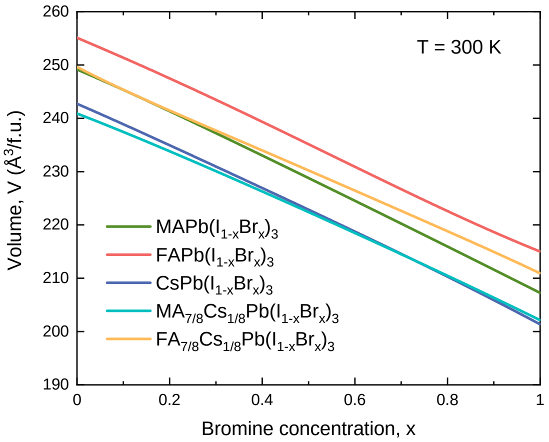


Supplementary Fig. 1. The equilibrium volume at room temperature per formula unit for the five compounds as a function of relative Br concentration $x$.

## SUPPLEMENTARY NOTE 2. BAND GAPS

The formulas for the halide composition-dependent band gaps, displayed in Supplementary Table 1, are given by

$$E_{\mathrm{g}}(x) = (1-x)E_{\mathrm{g}}(\mathrm{APbI_3}) + xE_{\mathrm{g}}(\mathrm{APbBr_3}) - bx(1-x), \tag{1}$$

where "A" is MA, FA, Cs, $MA_{7/8}Cs_{1/8}$, or $FA_{7/8}Cs_{1/8}$ and $b$ is the bowing parameter. The formulas for the band gaps of the single-cation compounds are obtained from experiment[3–5]. The band gaps of the pure I and pure Br double-cation compounds are obtained by a cation composition-weighted average of the single-cation compounds, using $E_{\mathrm{g}}(\mathrm{MAPbI_3}) = 1.57$ eV , $E_{\mathrm{g}}(\mathrm{MAPbBr_3}) = 2.29$ eV, $E_{\mathrm{g}}(\mathrm{FAPbI_3}) = 1.48$ eV, $E_{\mathrm{g}}(\mathrm{FAPbBr_3}) = 2.23$ eV, $E_{\mathrm{g}}(\mathrm{CsPbI_3}) = 1.77$ eV, and $E_{\mathrm{g}}(\mathrm{CsPbBr_3}) = 2.38$ eV. We set for simplicity $b(\mathrm{MA_{7/8}Cs_{1/8}}) = b(\mathrm{MA})$ and $b(\mathrm{FA_{7/8}Cs_{1/8}}) = b(\mathrm{FA})$.

Supplementary Table 1. Formulas for the band gap in eV as a function of relative Br concentration $x$ for the five compounds. The band gap plots are given in Fig. 2(b) of the main text.

| Compounds | Band gap |
|---|---|
| $MAPb(I_{1-x}Br_x)_3$ | $1.57(1-x) + 2.29x - 0.33x(1-x)$ |
| $FAPb(I_{1-x}Br_x)_3$ | $1.48(1-x) + 2.23x - 0.15x(1-x)$ |
| $CsPb(I_{1-x}Br_x)_3$ | $1.77(1-x) + 2.38x - 0.35x(1-x)$ |
| $MA_{7/8}Cs_{1/8}Pb(I_{1-x}Br_x)_3$ | $1.60(1-x) + 2.30x - 0.33x(1-x)$ |
| $FA_{7/8}Cs_{1/8}Pb(I_{1-x}Br_x)_3$ | $1.52(1-x) + 2.25x - 0.15x(1-x)$ |

## SUPPLEMENTARY NOTE 3. FINITE ELECTRON AND HOLE DIFFUSION LENGTHS

We show here that, despite the fact that Eqs. (1)-(3) in the main text are formally applicable only to the case of infinite electron and hole diffusion lengths, they can be applied to the initial stage of halide segregation also in the case of finite diffusion lengths.

Just before nucleation of a phase with Br concentration $x_2$ from a phase with Br concentration $x$, the photocarrier distribution is homogeneous with a density $n$ that is a solution of the equation $G = n/\tau + kn^2/V$ (see Eq. (3) in the main text). In the initial stage of nucleation, the nuclei will be much smaller than the diffusion lengths of electrons and holes, so that the photocarrier distribution within the nuclei will be homogeneous, with a constant density $n_2$. The volume fraction of the nucleated phase is $\delta\phi \equiv \phi_2$. The photocarrier density in the parent phase $n_1(\vec{r})$ after nucleation will not be homogeneous in the case of finite diffusion lengths. Instead of Eq. (3), which is only generally valid when the diffusion lengths are infinite, we now have:

$$G = \frac{1}{V_{\text{tot}}}\int_{V_1}\left(n_1(\vec{r})/\tau + kn_1^2(\vec{r})/V\right)d\vec{r} + \delta\phi(n_2/\tau + kn_2^2/V), \tag{2}$$

where $V_{\text{tot}}$ is the total volume, and the integral is performed over the volume $V_1$ of the parent phase. We write $n_1(\vec{r}) = n + \delta n(\vec{r})$, where $\delta n(\vec{r})$ is a function that scales linearly with $\delta\phi$ in the limit $\delta\phi \to 0$ (where all nuclei in the volume $V_{\text{tot}}$ are shrunk by an equal amount). Up to linear order in $\delta\phi$ we then have

$$G = (1-\delta\phi)(n/\tau + kn^2/V) + (1/\tau + 2nk/V)\frac{1}{V_{\text{tot}}}\int_{V_1}\delta n(\vec{r})d\vec{r} + \delta\phi(n_2/\tau + kn_2^2/V). \tag{3}$$

To linear order in $\delta\phi$ we can write, using Eq. (1) in the main text, $n_2 = \exp\left[-\left(E_{\text{g}}(x_2) - E_{\text{g}}(x)\right)/k_{\text{B}}T\right]n$. Since Eq. (3) holds for all values of the diffusion lengths, we conclude that the integral $\int_{V_1}\delta n(\vec{r})d\vec{r}$ is independent of the diffusion lengths. In particular, the integral is equal to its value for infinite diffusion lengths, hence

$$\frac{1}{V_{\text{tot}}}\int_{V_1}\delta n(\vec{r})d\vec{r} = \frac{1}{V_{\text{tot}}}\int_{V_1}(n_1(\vec{r}) - n)d\vec{r} = (1-\delta\phi)(n_1 - n) = \phi_1(n_1 - n), \tag{4}$$

where $n_1$ is the constant photocarrier density in the parent phase for infinite diffusion lengths. Inserting this in Eq. (3), we conclude that Eq. (3) in the main text is also valid in the case of finite diffusion lengths when applied to the initial stage of nucleation, where $\phi_2$ is infinitesimally small.

Equivalently, in the case of an inhomogeneous photocarrier density $n_1(\vec{r})$ in the parent phase due to finite diffusion lenghts, we have, instead of Eq. (2) in the main text,

$$\Delta F^{\star}(x_1, x_2, \phi_1, \phi_2, T) = \phi_1\Delta F(x_1, T) + \phi_2\Delta F(x_2, T) + \left[\frac{1}{V_{\text{tot}}}\int_{V_1} d\vec{r} n_1(\vec{r})\right]E_{\text{g}}(x_1) + n_2\phi_2 E_{\text{g}}(x_2), \tag{5}$$

which, by virtue of Eq. (4), is equal to Eq. (2) in the initial stage of nucleation. The conclusion is that, despite the fact that Eqs. (1)-(3) in the main text are formally only applicable to the case of infinite diffusion lengths of electrons and holes, they can be applied to the initial stage of halide segregation also in the case of finite diffusion lengths.

## SUPPLEMENTARY NOTE 4. RESULTS FOR $MA_{3/4}Cs_{1/4}Pb(I_{1-x}Br_x)_3$

In addition to $MA_{7/8}Cs_{1/8}Pb(I_{1-x}Br_x)_3$ (see main text) we performed calculations for $MA_{3/4}Cs_{1/4}Pb(I_{1-x}Br_x)_3$; see Supplementary Fig. 2. The lowest-enthalpy structure of $MA_{3/4}Cs_{1/4}Pb(I_{0.5}Br_{0.5})_3$ is shown in Supplementary Fig. 2a, where the two Cs cations in the $2 \times 2 \times 2$ supercell are taken at diagonal positions to profit as much from the remaining symmetry. Supplementary Figs. 2b and c show the enthalpy and free energy, which should be compared to Figs. 1i and n in the main text for $MA_{7/8}Cs_{1/8}Pb(I_{1-x}Br_x)_3$. Supplementary Figs. 2d-f show the phase diagrams in the dark and for two different photocarrier densities, which should be compared to Figs. 3d, i, and n. The results for the photocarrier density in Supplementary Figs. 2g-i (dashed lines) are compared to those for $MA_{7/8}Cs_{1/8}Pb(I_{1-x}Br_x)_3$ (solid lines) taken from Fig. 4.

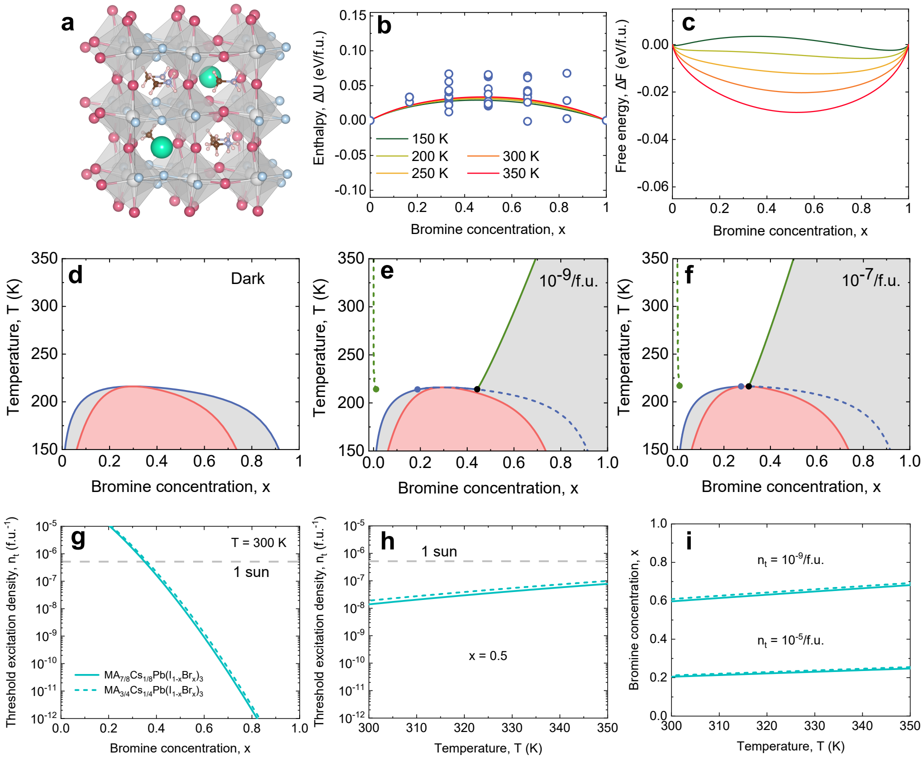


Supplementary Fig. 2. Results for $MA_{3/4}Cs_{1/4}Pb(I_{1-x}Br_x)_3$. **(a)** Atomic structure of the most stable configuration at relative Br concentration $x = 0.5$. The Cs cations are taken at diagonal positions in the $2 \times 2 \times 2$ supercell. **(b)** Mixing enthalpy as a function of Br concentration $x$. Circles: values calculated for each mixed configuration. Filled circle: value for the most stable configuration at $x = 0.5$, displayed in (a). Curves: results for the quasi-chemical approximation (QCA) at different temperatures. **(c)** Free energy. **(d)-(f)** Phase diagrams in the dark and at two different photocarrier densities. **(g)** Threshold photocarrier density $n_t$ at $T = 300$ K for light-induced halide segregation, as a function of Br concentration. **(h)** Threshold photocarrier density as a function of temperature for $x = 0.5$. The horizontal dashed line in (g) and (h) indicates the photocarrier density $n = 5 \times 10^{-7}$/f.u. in the mixed phase for about 1 sun illumination. **(i)** Bromine concentration as a function of temperature at threshold photocarrier densities $n_t = 10^{-9}$ and $10^{-5}$/f.u. The dashed lines in (g)-(i) show the results for $MA_{3/4}Cs_{1/4}Pb(I_{1-x}Br_x)_3$ and the solid lines those for $MA_{7/8}Cs_{1/8}Pb(I_{1-x}Br_x)_3$, taken from Fig. 4 in the main text.

## Supplementary References

[1] Shannon, R. D. Revised effective ionic radii and systematic studies of interatomic distances in halides and chalcogenides. *Acta crystallogr., sect. A: cryst. phys., difff., theor. gen. crystallogr.* **32**, 751–767 (1976).

[2] Ferdani, D. W. *et al.* Partial cation substitution reduces iodide ion transport in lead iodide perovskite solar cells. *Energy Environ. Sci.* **12**, 2264–2272 (2019).

[3] Noh, J. H., Im, S. H., Heo, J. H., Mandal, T. N. & Seok, S. I. Chemical management for colorful, efficient, and stable inorganic-organic hybrid nanostructured solar cells. *Nano Lett.* **13**, 1764–1769 (2013).

[4] Eperon, G. E. *et al.* Formamidinium lead trihalide: A broadly tunable perovskite for efficient planar heterojunction solar cells. *Energy Environ. Sci.* **7**, 982–988 (2014).

[5] Beal, R. E. *et al.* Cesium Lead Halide Perovskites with Improved Stability for Tandem Solar Cells. *J. Phys. Chem. Lett.* **7**, 746–751 (2016).
